# Effects of metformin on parasitological, pathological changes in the brain and liver and immunological aspects during visceral toxocariasis in mice

**DOI:** 10.1007/s00436-023-08011-1

**Published:** 2023-10-24

**Authors:** Amina M. Salama, Rasha A. Elmahy, Hoda A. Ibrahim, Alaa Ibrahim Mohamed Amer, Asmaa Fawzy Eltantawy, Dina I. Elgendy

**Affiliations:** 1 Medical Parasitology Department, Faculty of Medicine, Tanta University, Tanta, Egypt; 2 Zoology Department, Faculty of Science, Tanta University, Tanta, 31527 Egypt; 3 Medical Biochemistry Department, Faculty of Medicine, Tanta University, Tanta, Egypt; 4 Pathology Department, Faculty of Medicine, Tanta University, Tanta, Egypt; 5 Medical Pharmacology Department, Faculty of Medicine, Tanta University, Tanta, Egypt

**Keywords:** *Toxocara canis*, Albendazole, Metformin, MMP-9, Oxidative stress, FGF21, TNF-α, IL-12, IFN-γ, IL-4, IL-10; TGF-β

## Abstract

There are currently insufficient anthelmintic medications available for the treatment of toxocariasis. For instance, Albendazole (ABZ) is the preferred medication, but its effectiveness against tissue-dwelling parasites is limited. In addition, Metformin (MTF) is a widely used oral antidiabetic medication that is considered to be safe for treatment. This study aimed to investigate any potential effects of MTF, alone or in combination with ABZ, on mice infections caused by *Toxocara canis* (*T. canis*). The efficacy of the treatment was assessed in the acute and chronic phases of the infection by larval recovery and histopathological, immunohistochemical, and biochemical studies. The results showed that combined therapy significantly reduced larval counts in the liver, brain, and muscles and ameliorated hepatic and brain pathology. It reduced oxidative stress and TGF-β mRNA expression and increased FGF21 levels in the liver. It decreased TNF-α levels and MMP-9 expression in the brain. In addition, it increased serum levels of IL-12 and IFN-γ and decreased serum levels of IL-4 and IL-10. In the acute and chronic phases of the infection, the combined treatment was more effective than ABZ alone. In conclusion, this study highlights the potential role of MTF as an adjuvant in the treatment of experimental *T. canis* infection when administered with ABZ.

## Introduction

Human toxocariasis is a chronic systemic parasitic infection with a worldwide distribution mainly caused by *T. canis* larvae (Ma et al. 2018). Humans may contract the infection either by unintentionally swallowing embryonated *T. canis* eggs (Błaszkowska et al. 2015) or by ingesting viable *T. canis* second-stage larvae present in raw or undercooked meat from other paratenic hosts (Nijsse et al. 2016). Larvae can persist in paratenic hosts, such as humans, for extended periods without undergoing further maturation and have the ability to infiltrate several parenchymal organs (Clinton et al. 2010).

The severity of the symptoms that arise during toxocariasis depends on how many larvae are in the tissues and where they are located (Maizels 2013). In addition to the direct damage caused by the parasite’s migration, a variety of factors contributes to the destruction of tissues that occurs in toxocariasis. Significant contributors to this damage include oxidative stress and the host's immune response. (Demirci et al. 2006; Othman et al. 2011; Resende et al. 2015; Waindok et al. 2019). Antioxidants and anti-inflammatory medications are therefore anticipated to aid in the host’s defense against these harmful factors (Rogerio et al. 2008; Sayiner et al. 2021).

There are currently inadequate anthelmintic drugs available for the treatment of human toxocariasis. For this purpose, ABZ is the drug of choice (Horton 2000; Magnaval et al. 2022). It is superior to other used drugs as it is widely available and has no significant side effects due to its administration in a short regimen. Moreover, it can cross the blood-brain barrier (Schneier and Durand 2011). However, its low bioavailability limits its effectiveness against tissue-dwelling parasites (Daniel-Mwambete et al. 2004; Rigter et al. 2004). Furthermore, Musa et al. (2011), Deshayes et al. (2016), and Kroten et al. (2018) demonstrated that its efficacy against *T. canis* infection is moderate. Consequently, it is crucial to investigate new or synergistic agents that may enhance the efficacy of ABZ against toxocariasis.

MTF is a bioavailable oral antidiabetic that is widely used and safe (Foretz et al. 2014). It is one of the derivatives of biguanides that was initially used in the treatment of malaria (Sweeney et al. 2003). The available evidence suggests that the utilization of this treatment has demonstrated positive outcomes in the management of various parasitic diseases, including schistosomiasis mansoni (Salama et al. 2021), cystic echinococcosis (Loos et al. 2017), experimental trichinellosis (Othman et al. 2016), leishmaniasis braziliensis (Lima et al. 2020), American trypanosomiasis, and African trypanosomiasis (Martínez-Flórez et al. 2020). It is also characterized by anti-oxidative stress (Othman et al. 2016), anti-fibrotic property (Salama et al. 2021), and anti-inflammatory property (Kim et al. 2014). The purpose of this study was to evaluate the efficacy of MTF in reducing the pathogenic effects of *T. canis* infection and MTF’s potential use as an adjuvant with ABZ for the treatment of toxocariasis.

## Material and methods

### Parasite

Adult female *T. canis* worms obtained from the small intestines of infected pups were dissected to isolate the eggs. According to Faz-López et al. (2013), the eggs were cultivated to produce the infective stage, the egg harboring the second-stage larva. Each mouse in the infected group was inoculated intragastrically with 1000 infectious *T. canis* eggs. Before use, the embryonated eggs’ viability was determined through a microscopic examination of larval movement (Barrera et al. 2010).

### Drugs

Albendazole (ABZ): (Alzental) suspension (Eipico, Egypt) which contains 100 mg/5 ml was used. Each mouse received 100 mg/kg daily via oral gavage for five consecutive days (Nassef et al. 2014). Metformin (MTF): (Glucophage) 1000 mg/tablet (Minipharm, Egypt) was used. It was received via oral gavages to each mouse in a dosage of 150 mg/kg diluted in water once daily for 15 successive days (Salama et al. 2021).

### Animals and experimental design

In this study, 50 male Swiss albino laboratory mice (6–8 weeks old, 20–25 g in weight) were used. The mice were kept in typical cages at a temperature of 25 ± 2 °C, were fed a standard diet, and had free access to water. All mice were exposed to a 7-day acclimation period prior to the experiment.

### Experimental design

The following groups of mice were used:

1. Negative control group includes Group (I) (10 mice) uninfected control group.
2. Acute toxocariasis groups (A) (5 mice each) include Group (A/II) acute infected untreated control group (acute positive control), Group (A/III) treated with ABZ alone starting on the 8th day PI. (acute ABZ), Group (A/IV) treated with MTF alone starting on the 8th day PI (acute MTF), and Group (A/V) treated with ABZ and MTF starting on the 8th day PI. MTF was given in parallel with ABZ and then continued for the following ten days (acute combined).
3. Chronic toxocariasis groups (C) (5 mice each) include Group (C/II) chronic infected untreated control group (chronic positive control), Group (C/III) treated with ABZ alone starting on the 71st day PI (chronic ABZ), and Group (C/IV) treated with MTF alone starting on the 71st day PI (chronic MTF), and Group (C/V) treated with ABZ and MTF starting on the 71st day PI. MTF was given in parallel with ABZ and then continued for the following 10 days (chronic combined).

All mice of groups (A/II, A/III, A/IV, and A/V) and 5 mice from group (I) were sacrificed on the 22nd day PI, and all mice of groups (C/II, C/III, C/IV, and C/V) and 5 mice of group (I) were sacrificed on the 85th day PI. In order to do a parasitological investigation on all infected mice, larvae were counted in half of the liver, half of the brain, and muscles. For histopathological and immunohistochemical tests, identical brain and liver samples from each mouse were taken and stored in 10% formalin. Samples of each mouse’s brain, liver, and serum were collected and stored at −80 °C for biochemical analysis.

### Larval burden recovery

According to Liao et al. (2008), larval counting was done with some modifications. Each infected mouse’s liver and brain were weighed and homogenized separately in 1 ml of saline using a mortar and pestle. The homogenized liver, homogenized brain, and whole-body muscles of each mouse were then separately digested for 3 hours at 37°C in artificial digestive juice containing 1% pepsin (activity 1:10000) (w/v) and 1% concentrated HCL (v/v) in warm tap water. The mixtures were centrifuged (at 250 g for 10 min), and sedimented larvae were counted using a Mc. Master counting chamber. The percentage of reduction in the larval counts in the liver, brain, and muscles of mice was calculated using the following formula:

Percentage of reduction = 100 × (NC − NT)/NC

NC is the mean larval count in the corresponding control group/mouse; NT is the mean larval count in the treated group/mouse.

### Histopathological study

Tissue samples from the studied groups were fixed by 10% formalin, and then routine histologic processing was done by embedding in paraffin blocks, cutting by microtome, and staining with hematoxylin and eosin. Histopathological changes were observed in the study groups’ liver and brain. Granuloma formation was examined in hepatic tissues by counting the number and calculating their mean diameter. In each section, granulomas were counted in 10 high-power fields (×400) in 3 different fields per mouse to calculate the average. The largest diameter was measured using the ImageJ software (https://eliceirilab.org/software/imagej/). The mean diameter of granulomas per hepatic section was calculated (Klockiewicz et al. 2019).

### Immunohistochemical study

Matrix metalloproteinase-9 (MMP-9) expression was assessed by immunohistochemistry in accordance with the manufacturer’s instructions using the ultra-vision detection Kit (TP-015-HD, Lab Vision, USA) and rabbit polyclonal anti-MMP-9 (Cat. No. ab38898; Abcam, USA).

### Interpretation of MMP-9 positivity

A brown granular cytoplasmic stain was identified as MMP-9 positive immunostaining. The number of stained cells and the degree of staining were taken into account when calculating the score. Scores for staining intensity ranged from 0 to 3. 0 signified no staining, 1 slight staining, 2 moderate staining, and 3 high staining. On a scale of 1 to 4, the frequency of stained cells was rated as follows: 1 = (0–25%), 2 = (26–50%), 3 = (51–75%), and 4 = (75–100%). The intensity score and frequency score were multiplied to produce the final score. According to the final score, the staining was divided into five categories: score 0, absent expression; score +1 (1–2); score +2 (3–4); score +3 (6–8); and score +4 (9–12) (Niu et al. 2018).

### Biochemical study

Spectrophotometric measurements of nitric oxide (NO) (CAT# NO 25 33), Malondialdehyde (MDA) (CAT# MD 25 29), superoxide dismutase (SOD) (CAT# SD 25 21), and catalase (CAT# CA 25 17) levels were determined in liver tissue homogenate using kits provided by Bio-diagnostic (Giza, Egypt). Using ELISA kits, the levels of liver homogenate fibroblast growth factor 21 (FGF21) (CAT# ab212160, Abcam, USA), brain homogenate tumor necrosis factor-alpha (TNF-α) (CAT# ab208348, Abcam, USA), serum interleukin-12 (IL-12) (CAT# BMS6004, Thermo Fisher Scientific, USA), serum interferon-gamma (IFN-γ) (CAT# E-EL-M0048, Elabscience, USA), serum interleukin-4 (IL-4) (CAT# BMS613, Thermo Fisher scientific, USA), and serum interleukin-10 (IL-10) (CAT# OKBB00194, Aviva Systems Biology, USA) were determined. All ELISA techniques were read using a microplate reader (Stat Fax®2100, Fisher Bio block Scientific, France) at 450 nm with a correction wavelength set at 570 nm per the manufacturer’s recommendations.

### Real-time PCR analysis of liver homogenate relative transforming growth factor β (TGF β) mRNA expression

RNA was extracted from frozen liver homogenate using a Qiagen RNeasy Total RNA isolation kit (Qiagen, Hiden, Germany) and following the manufacturer’s instructions. The first strand of DNA was synthesized using the Super-Script III First-Strand Synthesis System for a real-time PCR kit (Life Technologies, Carlsbad, California, USA) and following the manufacturer’s instructions. PCR reactions were conducted using the Power SYBR Green PCR Master Mix (Life Technologies, Carlsbad, California, USA) according to the manufacturer’s instructions. The TGF-mRNA transcript amount was measured utilizing the housekeeping gene TGF-β as an internal control. These sequence-specific primers were developed as follows: mice TGF-β (Gene Bank accession NM_011577.2): up-stream: 5′-CTCTCTGCTCCTCCCTGTTCTA-3′, down-stream: 5′-ATAGATGGCGTTGTTGCGGT-3′ with amplicon size 202 bp, mice β-actin (Gene Bank accession NM_007393.5): up-stream: 5′-TTACAGGAAGTCCCTCACCC-3′, and down-stream: 5′-ACACAGAAGCAATGCTGTCAC-3′ with amplicon size 110 bp. Relative gene expression was calculated automatically by using the comparative threshold (Ct) method for the values of the target and the reference genes using the 2-ΔΔCT formula (Guo et al. 2021).

### Statistical analysis

The data were displayed as means ± standard deviation. One-way ANOVA was used to compare more than two groups, and the post hoc test was used to find the likelihood of significant differences between the dual means of the groups. The chi-square test was used to compare qualitative data between the two groups. Values were considered significant when *P* < 0.05. Statistical Program of Social Sciences (SPSS), software for Windows, version 18.0, was used to perform the statistical analyses.

## Results

### T. canis larvae counts in the liver, brain, and muscles

At both time points PI, the mean number of larvae detected in the brain and muscles of the treated groups was significantly lower than that of the positive control group. The combined group had the best results, with reduction percentages in the brain (62.0% and 58.0%) and muscles (79.7% and 73.2%) during the acute and chronic phases of infection, respectively. The combined treatment was significantly more effective than each drug administered separately. In the acute phase of infection, either ABZ or MTF alone significantly decreased liver larval counts. Nevertheless, neither drug significantly reduced the number of hepatic larvae in the chronic phase of the infection. The combined treatment was successful in reducing the number of larvae in the liver, with reduction percentages of 82.6% and 84.6% in the acute and chronic stages, respectively (Table 1).

**Table 1:** *T. canis* larval burden in the liver, brain, and muscles of mice in the infected groups

Larval counts/mouse		Groups (*n*=5)		Mean ± SD	Percentage of reduction	*F* test	Post hoc test	
In the liver	**Acute toxocariasis**	G A/II	Acute positive control	47 ± 7.38		89.929	***P1*** 0.001* ***P2*** 0.001* ***P3*** 0.001* ***P4*** 0.002* ***P5*** 0.001* ***P6*** 0.001* *P7* 0.222 *P8* 0.293	***P9*** 0.001* *P10* 0.860 ***P11*** 0.012* ***P12*** 0.008* ***P13*** 0.001* ***P14*** 0.001* ***P15*** 0.001* ***P16*** 0.006*
		G A/III	Acute ABZ	21.4 ± 3.44	54.5%			
		G A/IV	Acute MTF	29 ± 3.16	38.3%			
		G A/V	Acute combined	8.2 ± 0.84	82.6%			
	**Chronic toxocariasis**	G C/II	Chronic positive control	10.4 ± 3.65				
		G C/III	Chronic ABZ	7.6 ± 2.70	26.9%			
		G C/IV	Chronic MTF	8.0 ± 1.58	23.08%			
		G C/V	Chronic combined	1.6 ± 0.89	84.6%			
In the brain	**Acute toxocariasis**	G A/II	Acute positive control	123.00 ± 7.31		182.764	***P1*** 0.001* ***P2*** 0.001* ***P3*** 0.001* ***P4*** 0.001* ***P5*** 0.001* ***P6*** 0.001* ***P7*** 0.001* ***P8*** 0.001*	***P9*** 0.001* ***P10*** 0.001* ***P11*** 0.001* ***P12*** 0.001* ***P13*** 0.001* ***P14*** 0.001* ***P15*** 0.001* ***P16*** 0.001*
		G A/III	Acute ABZ	70.00 ± 4.36	43.1%			
		G A/IV	Acute MTF	86.80 ± 9.09	29.4%			
		G A/V	Acute combined	46.80 ± 5.17	62.0%			
	**Chronic toxocariasis**	G C/II	Chronic positive control	168.60 ± 6.80				
		G C/III	Chronic ABZ	120.20 ± 7.66	28.8%			
		G C/IV	Chronic MTF	140.60 ± 7.23	16.7%			
		G C/V	Chronic combined	70.80 ± 5.89	58.0%			
In the muscles	**Acute toxocariasis**	G A/II	Acute positive control	272.2 ± 16.69		209.666	***P1*** 0.001* ***P2*** 0.001* ***P3*** 0.001* ***P4*** 0.001* ***P5*** 0.001* ***P6*** 0.001* ***P7*** 0.001* ***P8*** 0.001*	***P9*** 0.001* ***P10*** 0.001* ***P11*** 0.001* ***P12*** 0.001* ***P13*** 0.001* ***P14*** 0.001* ***P15*** 0.001* ***P16*** 0.001*
		G A/III	Acute ABZ	144.2 ± 12.87	47%			
		G A/IV	Acute MTF	180 ± 12.79	33.9%			
		G A/V	Acute combined	55.4 ± 9.91	79.7%			
	**Chronic toxocariasis**	G C/II	Chronic positive control	323.8 ± 15.59				
		G C/III	Chronic ABZ	202.6 ± 19.84	37.4%			
		G C/IV	Chronic MTF	251.6 ± 13.24	22.3%			
		G C/V	Chronic combined	86.8 ± 10.26	73.2%			

***P1***: G A/II vs G A/III; ***P2***: G A/II vs G A/IV; ***P3***: G A/II vs G A/V; ***P4***: G A/III vs G A/IV; ***P5***: G A/III vs G A/V; ***P6***: G A/IV vs G A/V; ***P7***: G C/II vs G C/III; ***P8***: G C/II vs G C/IV; ***P9***: G C/II vs G C/V; ***P10***: G C/III vs G C/IV; ***P11***: G C/III vs G C/V; ***P12***: G C/IV vs G C/V; ***P13***: G A/II vs G C/II; ***P14***: G A/III vs G C/III; ***P15***: G A/IV vs G C/IV; ***P16***: G A/V vs G C/V

*P < 0.05 significant

*n*= number of studied mice in each group

### Histopathological and immunohistochemical results

#### Histopathological findings in the liver

In comparison to the positive control group, the mean number of granulomas per hepatic section decreased significantly in all ABZ-treated and MTF-treated groups. However, the difference between acute ABZ (G A/III) and acute MTF (G A/IV) groups was statistically insignificant. Furthermore, the combined groups significantly decreased when compared to the ABZ and MTF groups (Table 2).

**Table 2:** Comparison between the mean number and diameter of granulomas per hepatic section in```` different studied groups

Granulomas	Groups (*n*=5)		Mean ± SD	*F* test	Post hoc test	
Number	G A/II	Acute positive control	8.20 ± 0.84	36.513	***P1*** 0.001* ***P2*** 0.001* ***P3*** 0.001* *P4* 0.119 ***P5*** 0.001* ***P6*** 0.032*	***P9*** 0.001* ***P10*** 0.032* ***P11*** 0.001* ***P12*** 0.003* ***P13*** 0.001* ***P14*** 0.032* *P15* 0.119 *P16* 0.526
	G A/III	Acute ABZ	4.60 ± 1.14			
	G A/IV	Acute MTF	3.60 ± 1.14			
	G A/V	Acute combined	2.20 ± 0.45			
	G C/II	Chronic positive control	9.80 ± 1.10		***P7*** 0.001* ***P8*** 0.001*	
	G C/III	Chronic ABZ	6.00 ± 1.00			
	G C/IV	Chronic MTF	4.60 ± 0.89			
	G C/V	Chronic combined	2.60 ± 1.14			
Diameter	G A/II	Acute positive control	483.00 ± 42.71	26.165	***P1*** 0.001* ***P2*** 0.001* ***P3*** 0.001* *P4* 0.808 ***P5*** 0.006* ***P6*** 0.012*	***P9*** 0.001* *P10* 0.436 ***P11*** 0.002* ***P12*** 0.016* ***P13*** 0.001* *P14* 0.231 *P15* 0.504 *P16* 0.436
	G A/III	Acute ABZ	285.80 ± 73.28			
	G A/IV	Acute MTF	277.60 ± 60.87			
	G A/V	Acute combined	188.40 ± 49.55			
	G C/II	Chronic positive control	530.40 ± 53.80		***P7*** 0.001* ***P8*** 0.001*	
	G C/III	Chronic ABZ	326.60 ± 63.89			
	G C/IV	Chronic MTF	300.20 ± 33.72			
	G C/V	Chronic combined	214.80 ± 29.76			

***P1***: G A/II vs G A/III; ***P2***: G A/II vs G A/IV; ***P3***: G A/II vs G A/V; ***P4***: G A/III vs G A/IV; ***P5***: G A/III vs G A/V; ***P6***: G A/IV vs G A/V; ***P7***: G C/II vs G C/III; ***P8***: G C/II vs G C/IV; ***P9***: G C/II vs G C/V; ***P10***: G C/III vs G C/IV; ***P11***: G C/III vs G C/V; ***P12***: G C/IV vs G C/V; ***P13***: G A/II vs G C/II; ***P14***: G A/III vs G C/III; ***P15***: G A/IV vs G C/IV; ***P16***: G A/V vs G C/V

*P < 0.05 significant

*n*= number of studied mice in each group

There was a significant decrease in the mean diameter of hepatic granulomas in all treated groups compared to the matching positive control group. Compared to other treated groups, the combined group demonstrated significant decreases. The differences between each drug-treated group in the acute phase of the infection and its corresponding group in the chronic phase were not statistically significant (Table 2).

**Fig. 1:**
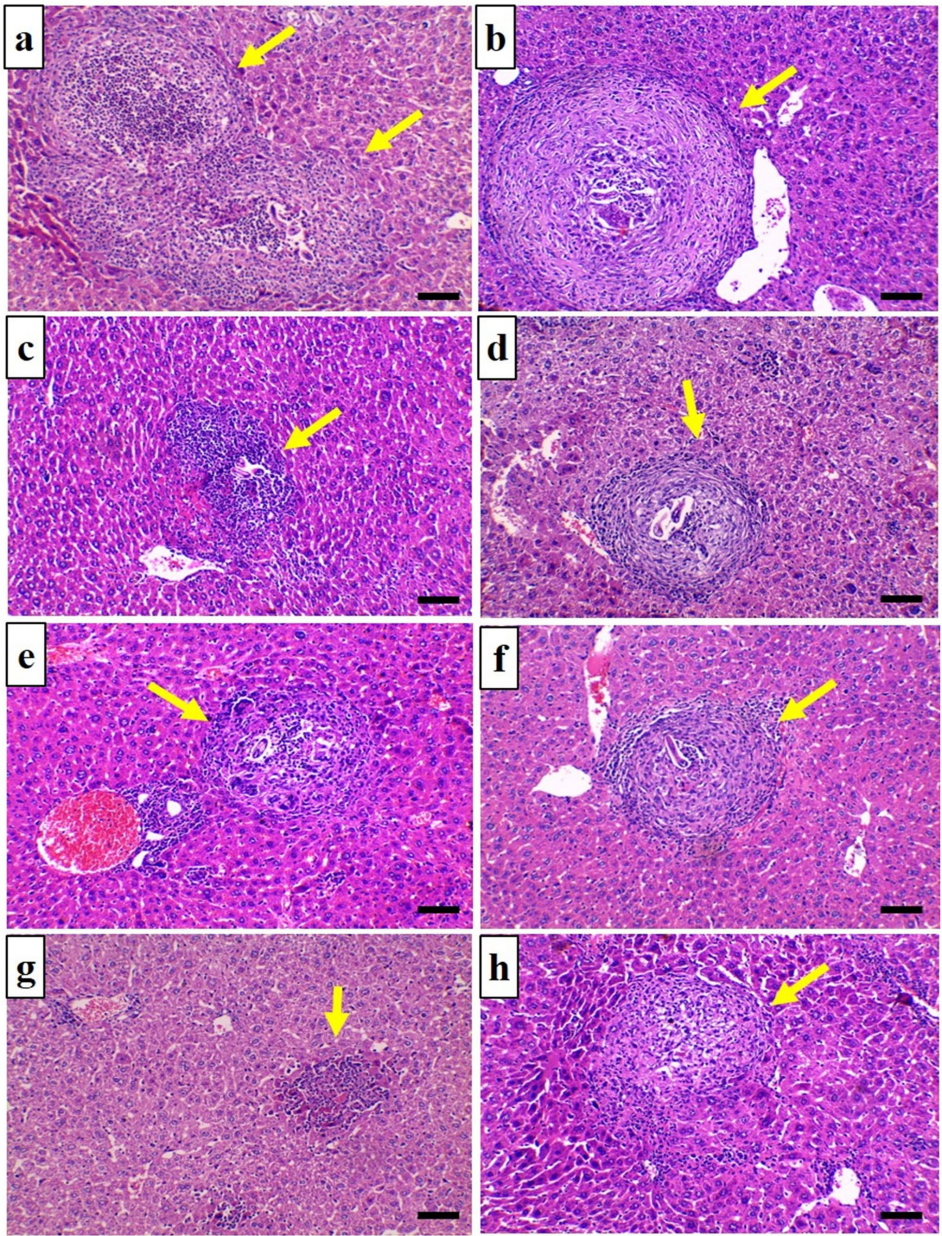
Hepatic sections (H&E x 200). **a** G A/II showed multiple cellular granulomas around T canis larvae. **b** G C/II group showed fibro cellular granulomas around T canis larvae. **c** G A/III showed a decrease in granuloma size and number, granulomas of cellular type. **d** G C/III showed a small fibro cellular granuloma. **e** G A/IV showed small cellular granuloma with a decrease in the number of granulomas. **f** G C/IV showed small fibro cellular granuloma with a decrease in the number of granulomas. **g** G A/V showed a tiny residual of cellular granuloma. **h** G C/V showed very small fibro cellular granuloma. Scale bar = 50 μm

Multiple cellular granulomas composed of a collection of inflammatory cells, including macrophages, lymphocytes, and eosinophils, were observed in liver sections of the acute positive control group (Fig. 1a). The chronic positive control group showed multiple fibro cellular granulomas with excessive fibrous tissue (Fig. 1b). Granulomas were examined from acute ABZ (Fig. 1c) and acute MTF (Fig. 1e); they were cellular granulomas. Chronic ABZ (Fig 1d) and chronic MTF (Fig. 1f) showed fibrocellular granulomas formed of inflammatory cells with scanty fibrous tissue. Granuloma resolution was observed in the combined-treatment groups (Fig. 1g, h).

#### Histopathological findings in the brain

**Fig. 2:**
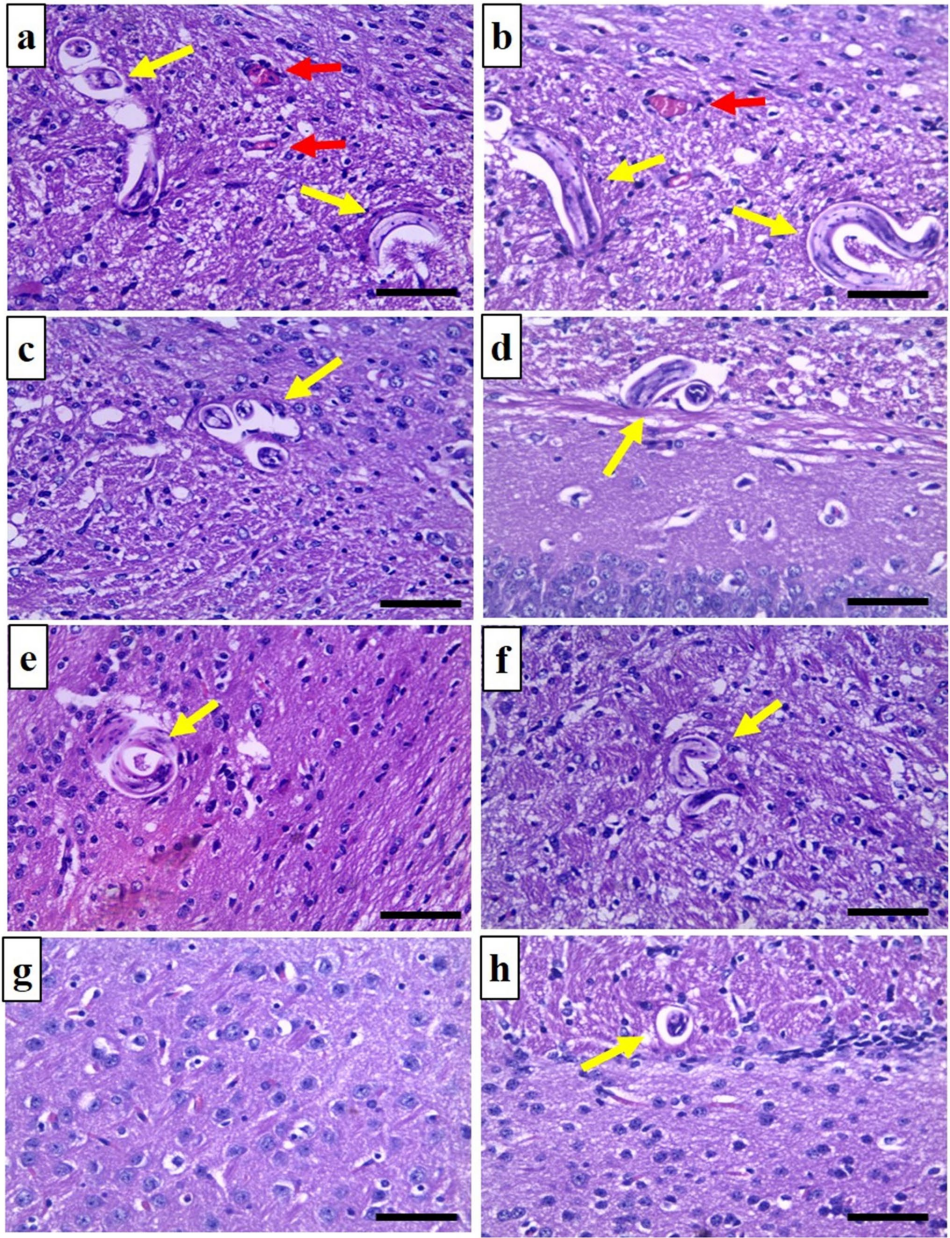
Brain sections (H&E × 400), **a** from G A/II showing scattered *T. canis* larvae (yellow arrows) with few congested blood vessels (red arrows), **b** from G C/II showing numerous longitudinal and transverse sections in *T. canis* larvae with no inflammatory cells around them, **c** from G A/III showing a decreased number of *T. canis* larvae and no obvious blood vessels congestion, **d** from G C/III showing similar findings as the G A/III, **e** from G A/IV showing a tangential section in a larva, **f** from G C/IV showing a longitudinal section in *T. canis* larva, **g** from G A/V showing no obvious larva, and **h** from G C/V showing a cross-section in *T. canis* larva. Scale bar = 50 μm

G A/II and G C/II brain sections revealed scattered *T. canis* larvae in parenchymal tissue with few congested blood vessels. Nonetheless, these larvae did not induce any surrounding inflammatory cell infiltration (Fig. 2a, b). Sections of ABZ-treated groups (G A/III and G C/III) and MTF-treated groups (G A/IV and G C/ IV) showed few larvae with no congested blood vessels. (Figure 2c–f). Sections of the acute combined group (G A/V) revealed no larvae with no congested blood vessels (Fig. 2g). The chronic combined group (G C/V) showed few larvae and no congestion in blood vessels (Fig. 2h).

#### Immunohistochemical study of the MMP-9 reaction in brain tissues

**Fig. 3:**
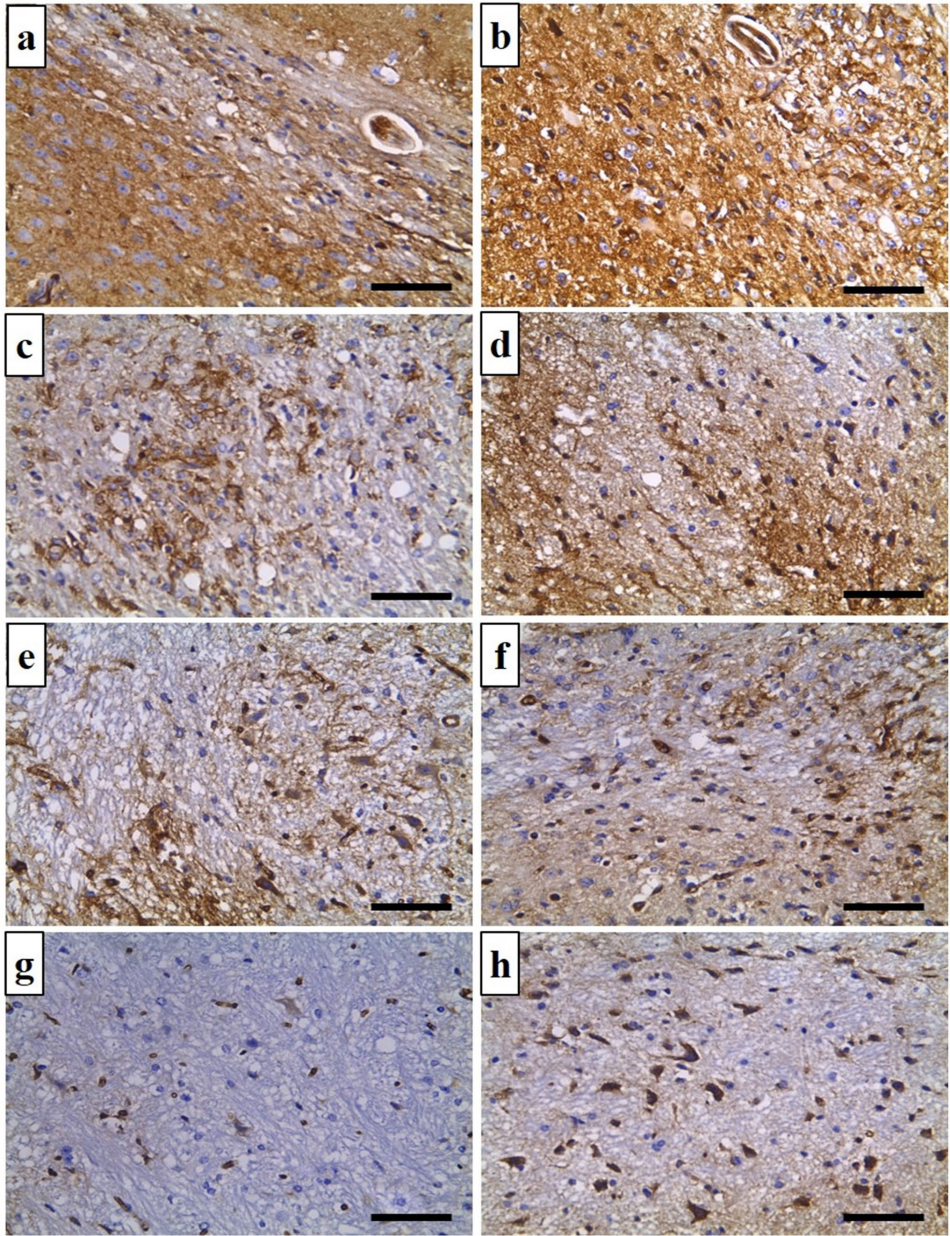
Brain sections immunohistochemical expression of MMP 9 (immunoperoxidase × 400): **a** G A/II showed high cytoplasmic expression in astrocytes and glial cells in brain parenchyma around *T. canis* larva (+3). **b** G C/II showed higher cytoplasmic expression in the brain parenchyma (+4). **c** G A/III showed mild cytoplasmic expression (+1). **d** G C/III showed moderate cytoplasmic expression(+2). **e** G A/IV showed mild expression in brain cells (+1). **f** G C/IV showed moderate cytoplasmic expression in cells brain (+2). **g** G A/V showed negative expression in brain cells (0). **h** G C/V showed mild cytoplasmic expression in cells (+1). Scale bar = 50 μm

The chronic positive control group (G C/II) had significantly higher MMP-9 reactivity in brain tissues than the acute positive control group (G A/II). In addition, its expression in the positive control groups (G A/II and G C/II) (Fig. 3a, b) was significantly higher than in the corresponding treated groups (Fig. 3c–h). ABZ-treated groups (Fig. 3c, d) and MTF-treated groups (Fig. 3e, f) exhibited decreased MMP-9 production at both time periods post-infection, with more pronounced effects seen during the acute phase rather than the chronic phase of the infection. The presence of MMP-9 reactivity was either not detected or found to be very limited in brain tissue obtained from the acute combined group (Fig. 3g). Similarly, the reactivity of MMP-9 was found to be minimal in the chronic combined group (Fig. 3h), as illustrated in Table 3.

**Table 3:** Immunohistochemical expression of MMP-9 in brain sections of studied groups

Groups (*n*= 5)		MMP-9						
		0	+1	+2	+3	+4	*X*^2^	*P* value
G A/II	Acute positive control	0	0	1	1	3	46.029	0.001*
G A/III	Acute ABZ	0	2	0	1	2		
G A/IV	Acute MTF	0	3	1	0	1		
G A/V	Acute combined	3	2	0	0	0		
G C/II	Chronic positive control	0	0	0	1	4		
G C/III	Chronic ABZ	0	1	1	1	2		
G C/IV	Chronic MTF	0	2	1	1	1		
G C/V	Chronic combined	3	1	1	0	0		

*P <0.05 (significant)

*n*= number of studied mice in each group

Chi-square (*X*^2^) test of significance

### Biochemical results

#### NO and redox status markers in the liver

G C/II group showed a more significant increment in levels of NO and MDA than the G A/II group as compared to the negative control (G I). All treated groups exhibited a significant decline in NO levels compared to the corresponding positive control group. However, the G C/V group showed a more substantial elevation in NO levels than all infected groups except G C/IV. MDA levels in all treated groups except G A/III were significantly lower than the matching positive control group, as depicted in Table 4.

**Table 4:** Levels (mean ± SD) of redox status markers in the liver of all studied mice

Parameter	Groups (*n*=5)		Mean ± SD	*F* test	Post hoc test	
NO (μmol/mg tissue)	G I	Negative control	12.23 ± 0.80	320.069	***P1*** 0.001* ***P2*** 0.001* ***P3*** 0.001* ***P4*** 0.001* ***P5*** 0.001* ***P6*** 0.001* ***P7*** 0.001* *P8* 0.482 ***P9*** 0.001* ***P10*** 0.001* ***P11*** 0.001* ***P12*** 0.001*	***P13*** 0.001* ***P14*** 0.016* ***P15*** 0.001* ***P16*** 0.001* ***P17*** 0.001* ***P18*** 0.001* ***P19*** 0.001* *P20* 0.093 ***P21*** 0.001* ***P22*** 0.001* ***P23*** 0.001* ***P24*** 0.001*
	G A/II	Acute positive control	30.07 ± 1.08			
	G A/III	Acute ABZ	21.88 ± 0.78			
	G A/IV	Acute MTF	18.61 ± 0.89			
	G A/V	Acute combined	16.99 ± 0.57			
	G C/II	Chronic positive control	35.03 ± 1.58			
	G C/III	Chronic ABZ	25.76 ± 1.27			
	G C/IV	Chronic MTF	13.79 ± 0.81			
	G C/V	Chronic combined	12.69 ± 0.93			
MDA (nmol/gm tissue)	G I	Negative control	36.28 ± 1.39	297.756	***P1*** 0.001* ***P2*** 0.001* ***P3*** 0.001* ***P4*** 0.001* ***P5*** 0.001* ***P6*** 0.001* ***P7*** 0.020* ***P8*** 0.025* *P9* 0.211 ***P10*** 0.001* ***P11*** 0.001* ***P12*** 0.001*	***P13*** 0.001* ***P14*** 0.001* ***P15*** 0.001* ***P16*** 0.001* ***P17*** 0.001* ***P18*** 0.001* ***P19*** 0.001* ***P20*** 0.001* ***P21*** 0.001* ***P22*** 0.001* ***P23*** 0.001* ***P24*** 0.001*
	G A/II	Acute positive control	63.85 ± 1.30			
	G A/III	Acute ABZ	62.56 ± 1.02			
	G A/IV	Acute MTF	47.83 ± 0.69			
	G A/V	Acute combined	43.29 ± 1.53			
	G C/II	Chronic positive control	71.20 ± 2.49			
	G C/III	Chronic ABZ	55.09 ± 2.14			
	G C/IV	Chronic MTF	42.91 ± 1.98			
	G C/V	Chronic combined	38.66 ± 1.01			
SOD (U/gm tissue)	G I	Negative control	2.74 ± 0.23	220.059	***P1*** 0.001* ***P2*** 0.001* ***P3*** 0.001* ***P4*** 0.001* ***P5*** 0.001* ***P6*** 0.001* ***P7*** 0.020* ***P8*** 0.037* ***P9*** 0.001* ***P10*** 0.001* ***P11*** 0.001* ***P12*** 0.001*	***P13*** 0.001* ***P14*** 0.029* ***P15*** 0.001* ***P16*** 0.001* ***P17*** 0.001* ***P18*** 0.001* ***P19*** 0.001* *P20* 0.138 *P21* 0.454 ***P22*** 0.001* ***P23*** 0.001* ***P24*** 0.001*
	G A/II	Acute positive control	0.86 ± 0.05			
	G A/III	Acute ABZ	1.18 ± 0.07			
	G A/IV	Acute MTF	2.14 ± 0.11			
	G A/V	Acute combined	2.30 ± 0.08			
	G C/II	Chronic positive control	0.91 ± 0.03			
	G C/III	Chronic ABZ	1.46 ± 0.06			
	G C/IV	Chronic MTF	2.48 ± 0.04			
	G C/V	Chronic combined	2.58 ± 0.18			
Catalase (U/gm tissue)	G I	Negative control	37.49 ± 1.54	265.403	***P1*** 0.001* ***P2*** 0.001* ***P3*** 0.001* ***P4*** 0.001* ***P5*** 0.001* ***P6*** 0.001* *P7* 0.494 *P8* 0.145 ***P9*** 0.001* ***P10*** 0.001* ***P11*** 0.001* ***P12*** 0.001*	***P13*** 0.001* ***P14*** 0.001* ***P15*** 0.001* ***P16*** 0.001* ***P17*** 0.001* ***P18*** 0.001* ***P19*** 0.001* *P20* 0.494 ***P21*** 0.001* ***P22*** 0.001* ***P23*** 0.001* ***P24*** 0.002*
	G A/II	Acute positive control	15.75 ± 0.55			
	G A/III	Acute ABZ	24.58 ± 1.40			
	G A/IV	Acute MTF	29.66 ± 0.38			
	G A/V	Acute combined	34.11 ± 1.50			
	G C/II	Chronic positive control	18.71 ± 1.15			
	G C/III	Chronic ABZ	27.72 ± 1.23			
	G C/IV	Chronic MTF	37.01 ± 0.91			
	G C/V	Chronic combined	36.47 ± 0.60			

***P1***: G I vs G A/II; ***P2***: G I vs G A/III; ***P3***: G I vs G A/IV; ***P4***: G I vs G A/V; ***P5***: G I vs G C/II; ***P6***: G I vs G C/III; ***P7***: G I vs G C/IV; ***P8***: G I vs G C/V; ***P9***: G A/II vs G A/III; ***P10***: G A/II vs A/IV; ***P11***: G A/II vs G A/V; ***P12***: G A/III vs G A/IV; ***P13***: G A/III vs G A/V; ***P14***: G A/IV vs G A/V; ***P15***: G C/II vs G C/III; ***P16***: G C/II vs G C/IV; ***P17***: G C/II vs G C/V; ***P18***: G C/III vs G C/IV; ***P19***: G C/III vs G C/V; ***P20***: G C/IV vs G C/V; ***P21***: G A/II vs G C/II; ***P22***: G A/III vs G C/III; ***P23***: G A/IV vs G C/IV; ***P24***: G A/V vs G C/V

*P < 0.05 significant

*n*= number of studied mice in each group

G A/II and G C/II groups exhibited a more pronounced increase in the levels of SOD & catalase than the negative control Group (G I). Both SOD and catalase levels were significantly higher in the G A/IV and G A/V groups compared to the G A/III group. Similarly, both markers were significantly elevated in the G C/IV and G C/V groups relative to the G C/III group. In treated groups relative to G A/II and G C/II, the levels of SOD and catalase did not differ significantly between G C/IV and G C/V (Table 4).

### Levels of FGF21 in liver homogenate and TNF-α in brain homogenate

G A/II and G C/II had significantly lower levels of FGF21 compared to G I. All treated groups had significantly higher concentrations than the positive control groups. Additionally, levels of FGF21 in combined treated groups showed a significant increase in comparison with other treated groups. G A/II demonstrated a more significant increase in TNF-α levels than G C/II when compared to the negative control. However, G C/V demonstrated a higher decrease in TNF-α levels as compared to MTF- and ABZ-treated groups. TNF-α levels were decreased in G C/IV than in G A/IV. Moreover, G C/III had decreased levels of TNF-α than G A/III. There was a significant difference in TNF-α levels in all study groups (*P* < 0.001), as shown in Table 5.

**Table 5:** Levels (mean ± SD) of FGF21 in the liver and TNFα in the brain of all studied mice

Parameter	Groups (*n*=5)		Mean ± SD	F test	Post hoc test	
FGF21 (ng/mg tissue)	G I	Negative control	53.81 ± 0.99	615.933	***P1*** 0.020* ***P2*** 0.001* ***P3*** 0.001* ***P4*** 0.001* ***P5*** 0.001* ***P6*** 0.001* ***P7*** 0.001* ***P8*** 0.001* ***P9*** 0.001* ***P10*** 0.001* ***P11*** 0.001* ***P12*** 0.001*	***P13*** 0.001* ***P14*** 0.001* ***P15*** 0.001* ***P16*** 0.001* ***P17*** 0.001* ***P18*** 0.001* ***P19*** 0.001* ***P20*** 0.001* ***P21*** 0.006* ***P22*** 0.001* ***P23*** 0.001* ***P24*** 0.001*
	G A/II	Acute positive control	43.47 ± 0.85			
	G A/III	Acute ABZ	81.40 ± 0.90			
	G A/IV	Acute MTF	89.67 ± 0.45			
	G A/V	Acute combined	92.35 ± 0.94			
	G C/II	Chronic positive control	45.35 ± 1.91			
	G C/III	Chronic ABZ	65.11 ± 0.47			
	G C/IV	Chronic MTF	74.09 ± 1.12			
	G C/V	Chronic combined	88.85 ± 0.72			
TNFα (ng/mg tissue)	G I	Negative control	82.09 ± 0.78	812.109	***P1*** 0.001* ***P2*** 0.001* ***P3*** 0.001* ***P4*** 0.001* ***P5*** 0.001* ***P6*** 0.001* ***P7*** 0.001* ***P8*** 0.001* ***P9*** 0.001* ***P10*** 0.001* ***P11*** 0.001* ***P12*** 0.001*	***P13*** 0.001* ***P14*** 0.001* ***P15*** 0.001* ***P16*** 0.001* ***P17*** 0.001* ***P18*** 0.001* ***P19*** 0.001* ***P20*** 0.001* ***P21*** 0.001* ***P22*** 0.001* ***P23*** 0.001* ***P24*** 0.001*
	G A/II	Acute positive control	126.71 ± 1.09			
	G A/III	Acute ABZ	112.48 ± 0.72			
	G A/IV	Acute MTF	105.34 ± 0.51			
	G A/V	Acute combined	95.88 ± 0.85			
	G C/II	Chronic positive control	123.43 ± 1.15			
	G C/III	Chronic ABZ	101.74 ± 0.87			
	G C/IV	Chronic MTF	91.85 ± 0.90			
	G C/V	Chronic combined	84.64 ± 1.17			

***P1***: G I vs G A/II; ***P2***: G I vs G A/III; ***P3***: G I vs G A/IV; ***P4***: G I vs G A/V; ***P5***: G I vs G C/II; ***P6***: G I vs G C/III; ***P7***: G I vs G C/IV; ***P8***: G I vs G C/V; ***P9***: G A/II vs G A/III; ***P10***: G A/II vs A/IV; ***P11***: G A/II vs G A/V; ***P12***: G A/III vs G A/IV; ***P13***: G A/III vs G A/V; ***P14***: G A/IV vs G A/V; ***P15***: G C/II vs G C/III; ***P16***: G C/II vs G C/IV; ***P17***: G C/II vs G C/V; ***P18***: G C/III vs G C/IV; ***P19***: G C/III vs G C/V; ***P20***: G C/IV vs G C/V; ***P21***: G A/II vs G C/II; ***P22***: G A/III vs G C/III; ***P23***: G A/IV vs G C/IV; ***P24***: G A/V vs G C/V

*P < 0.05 significant

*n*= number of studied mice in each group

### Serum cytokine levels

IL-12 and IFN-γ levels showed significant decreases in G A/II and G C/II compared to G I. Treatment administration induced significant elevation in levels of both markers in comparison with G A/II and G C/II. The differences in levels of both markers were non-significant between G I and G A/V. Likewise, no significant change was detected in levels of IL-12 between G A/III and G A/V. Also, IFN-γ levels in G C/V did not differ significantly from those in G I (Table 6).

**Table 6:** Serum cytokines levels (mean ± SD) in all studied mice

Parameter	Groups (*n*=5)		Mean ± SD	*F* test	Post hoc test	
IL-12 pg/ml	G I	Negative control	399.65 **±** 16.42	272.360	***P1*** 0.001* ***P2*** 0.001* ***P3*** 0.001* *P4* 0.063 ***P5*** 0.001* ***P6*** 0.001* ***P7*** 0.001* ***P8*** 0.001* ***P9*** 0.001* ***P10*** 0.001* ***P11*** 0.001* ***P12*** 0.007*	***P13*** 0.052 ***P14*** 0.001* ***P15*** 0.001* ***P16*** 0.001* ***P17*** 0.001* ***P18*** 0.001* ***P19*** 0.001* ***P20*** 0.001* ***P21*** 0.001* ***P22*** 0.001* ***P23*** 0.001* ***P24*** 0.015*
	G A/II	Acute positive control	221.12 **±** 11.76			
	G A/III	Acute ABZ	371.91 **±** 12.48			
	G A/IV	Acute MTF	351.59 **±** 15.48			
	G A/V	Acute combined	386.10 **±** 10.34			
	G C/II	Chronic positive control	174.31 **±** 8.86			
	G C/III	Chronic ABZ	268.75 **±** 5.54			
	G C/IV	Chronic MTF	249.61 **±** 8.19			
	G C/V	Chronic combined	368.00 **±** 6.10			
IFN-γ pg/ml	G I	Negative control	53.61 **±** 8.25	36.420	***P1*** 0.001* ***P2*** 0.001* ***P3*** 0.001* *P4* 0.225 ***P5*** 0.001* ***P6*** 0.001* ***P7*** 0.001* *P8* 0.353 ***P9*** 0.002* ***P10*** 0.001* ***P11*** 0.001* ***P12*** 0.116	***P13*** 0.001* ***P14*** 0.009* ***P15*** 0.001* ***P16*** 0.001* ***P17*** 0.001* ***P18*** 0.043* ***P19*** 0.001* ***P20*** 0.001* ***P21*** 0.001* ***P22*** 0.004* ***P23*** 0.014* ***P24*** 0.001*
	G A/II	Acute positive control	29.38 **±** 3.95			
	G A/III	Acute ABZ	38.53 **±** 2.74			
	G A/IV	Acute MTF	42.85 **±** 3.20			
	G A/V	Acute combined	50.30 **±** 1.81			
	G C/II	Chronic positive control	19.85 **±** 3.43			
	G C/III	Chronic ABZ	30.33 **±** 2.53			
	G C/IV	Chronic MTF	35.94 **±** 3.87			
	G C/V	Chronic combined	51.09 **±** 4.87			
IL4 (pg/ml)	G I	Negative control	20.65 ± 0.56	751.245	***P1*** 0.001* ***P2*** 0.001* ***P3*** 0.001* ***P4*** 0.001* ***P5*** 0.001* ***P6*** 0.001* ***P7*** 0.001* ***P8*** 0.001* ***P9*** 0.001* ***P10*** 0.001* ***P11*** 0.001* ***P12*** 0.001*	***P13*** 0.001* ***P14*** 0.001* ***P15*** 0.001* ***P16*** 0.001* ***P17*** 0.001* ***P18*** 0.001* ***P19*** 0.001* ***P20*** 0.001* ***P21*** 0.001* ***P22*** 0.001* ***P23*** 0.001* ***P24*** 0.001*
	G A/II	Acute positive control	58.13 ± 1.31			
	G A/III	Acute ABZ	47.53 ± 0.93			
	G A/IV	Acute MTF	44.27 ± 1.01			
	G A/V	Acute combined	34.23 ± 1.43			
	G C/II	Chronic positive control	52.09 ± 0.55			
	G C/III	Chronic ABZ	41.32 ± 0.69			
	G C/IV	Chronic MTF	37.72 ± 0.69			
	G C/V	Chronic combined	24.13 ± 1.36			
IL-10 pg/ml	G I	Negative control	83.28 **±** 7.51	131.178	***P1*** 0.001* ***P2*** 0.001* ***P3*** 0.001* *P4* 0.424 ***P5*** 0.001* ***P6*** 0.001* ***P7*** 0.001* ***P8*** 0.022* ***P9*** 0.001* ***P10*** 0.001* ***P11*** 0.001* *P12* 0.277	***P13*** 0.001* ***P14*** 0.001* ***P15*** 0.001* ***P16*** 0.001* ***P17*** 0.001* ***P18*** 0.008* ***P19*** 0.001* ***P20*** 0.001* ***P21*** 0.001* ***P22*** 0.001* ***P23*** 0.001* *P24* 0.121
	G A/II	Acute positive control	146.71 **±** 6.17			
	G A/III	Acute ABZ	111.34 **±** 9.54			
	G A/IV	Acute MTF	116.88 **±** 5.57			
	G A/V	Acute combined	87.33 **±** 5.27			
	G C/II	Chronic positive control	210.74 **±** 7.70			
	G C/III	Chronic ABZ	141.05 **±** 5.48			
	G C/IV	Chronic MTF	155.05 **±** 14.83			
	G C/V	Chronic combined	95.30 **±** 3.66			

***P1***: G I vs G A/II; ***P2***: G I vs G A/III; ***P3***: G I vs G A/IV; ***P4***: G I vs G A/V; ***P5***: G I vs G C/II; ***P6***: G I vs G C/III; ***P7***: G I vs G C/IV; ***P8***: G I vs G C/V; ***P9***: G A/II vs G A/III; ***P10***: G A/II vs A/IV; ***P11***: G A/II vs G A/V; ***P12***: G A/III vs G A/IV; ***P13***: G A/III vs G A/V; ***P14***: G A/IV vs G A/V; ***P15***: G C/II vs G C/III; ***P16***: G C/II vs G C/IV; ***P17***: G C/II vs G C/V; ***P18***: G C/III vs G C/IV; ***P19***: G C/III vs G C/V; ***P20***: G C/IV vs G C/V; ***P21***: G A/II vs G C/II; ***P22***: G A/III vs G C/III; ***P23***: G A/IV vs G C/IV; ***P24***: G A/V vs G C/V

*P < 0.05 significant

*n*= number of studied mice in each group

In contrast, levels of IL-4 and IL-10 demonstrated a significant rise in G A/II and G C/II compared to G I. All treated groups had significantly lower levels than the corresponding positive control group. IL-10 levels did not differ significantly between G A/V and G I. During the acute and chronic phases of the infection, the levels of both IL-4 and IL-10 in the combined groups were significantly lower than those in the groups receiving either ABZ or MTF alone (Table 6).

### TGF β mRNA expression in liver homogenate

**Fig. 4:**
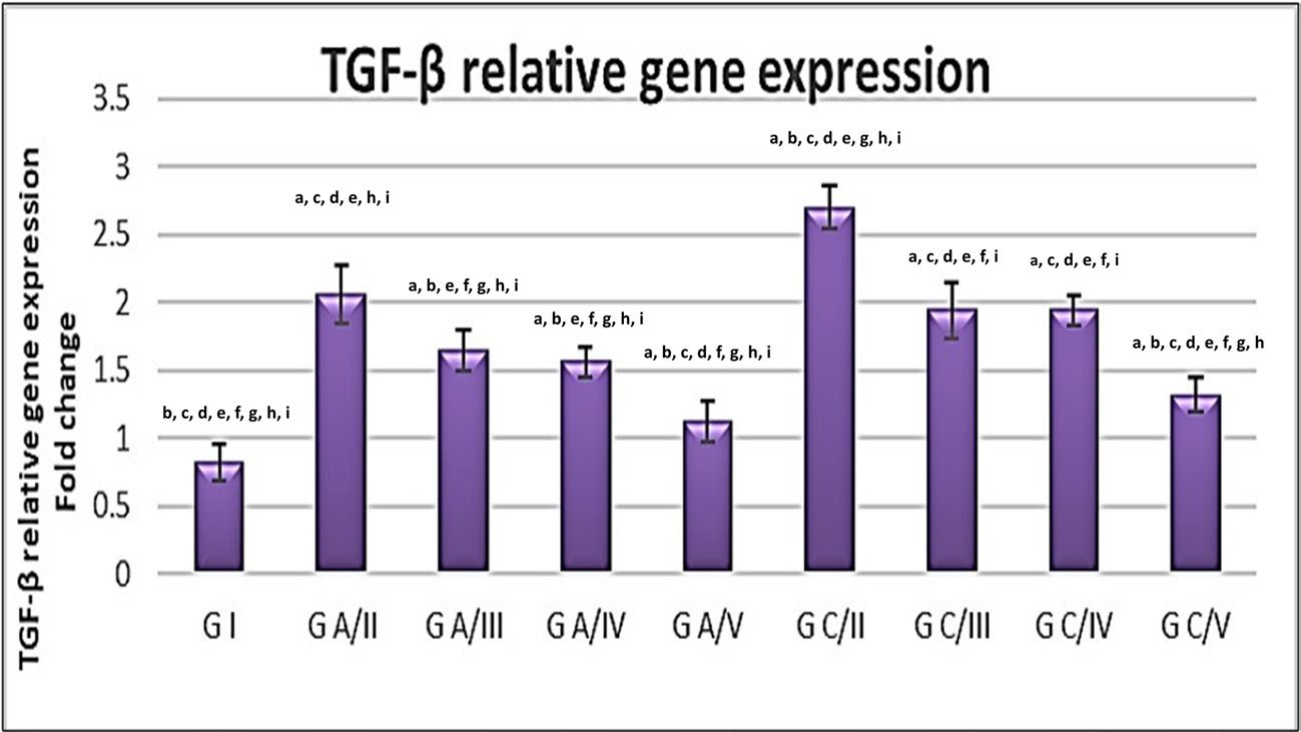
Relative TGF- β mRNA expression in liver tissue homogenates of different groups. vertical bar illustrates the means ± standard deviation. a indicates significant vs. G I, b indicates significant vs. G A/II, c indicates significant vs. G A/III, d indicates significant vs. G A/IV, e indicates significant versus G A/V, f indicates significant vs. G C/II, g indicates significant vs. G C/III, h indicates significant vs. G C/IV, and i indicates significant vs. G C/V

There were significant differences in relative liver TGF-β gene mRNA expression between all treatment groups and the corresponding positive control group (*P* < 0.001). There was a statistically significant increase in its expression in the chronic positive control group compared to other groups (*P* < 0.001). However, the acute combined group showed a significant decrease compared to other studied groups. There were non-significant differences between acute ABZ and acute MTF (*P* < 0.420) as well as between chronic ABZ and chronic MTF (*P* < 1.0) (Fig. 4).

## Discussion

Currently, the efficacy of the available anti-toxocariasis drugs remains suboptimal. ABZ has emerged as one of the recommended drugs (Magnaval et al. 2022). Nevertheless, its poor solubility and limited tissue absorption are factors that limit its efficacy (Márquez-Navarro et al. 2009). Accordingly, finding novel therapeutic agents or adjuvants that could enhance the efficacy of ABZ is appropriate for the treatment of visceral toxocariasis.

In the current study, combined treatment was more effective on *T. canis* larvae in the brains, livers, and muscles than administration of ABZ alone during the acute and chronic stages of the infection. Additionally, MTF-treated groups showed significant reductions compared to the positive control groups. To our knowledge, it is the first time to investigate the efficacy of MTF on toxocariasis. However, these results were not surprising as previous studies on the efficacy of MTF in the treatment of other parasitic infections reported its effectiveness in reducing parasite burdens. For instance, MTF could reduce the total number of larvae in the muscles of *Trichinella spiralis*-infected mice (Othman et al. 2016). In addition, levels of parasitemia in *Plasmodium yoelii*-infected mice were significantly reduced by MTF administration (Miyakoda et al. 2018). Furthermore, the synergistic effects of combined therapy of both ABZ and MTF were previously reported in the treatment of cystic echinococcosis, where the combined therapy induced a significant effect on both metacestodes and protoscoleces than ABZ alone (Loos et al. 2017).

The liver is one of the visceral organs most frequently impacted in visceral *T. canis* larval migration. Larvae actively moving through the liver generate tissue disorganization and granulomatous inflammation, which can result in granulomatous hepatitis (Kayes 1997). In terms of histopathological findings in the liver, the current study revealed that treatment of *T. canis*-infected mice with ABZ and MTF significantly decreased granuloma numbers and granuloma diameter and fibrosis compared to ABZ-treated groups. This observation was true for the infection’s acute and chronic phases. The decrease in granuloma counts paralleled the decrease in hepatic larval counts. Comparatively, the MTF-treated groups had significantly smaller granulomas than the positive control groups. Salama et al. (2021) demonstrated the efficacy of MTF in reducing the size of hepatic granuloma and ameliorating hepatic fibrosis in *Schistosoma mansoni*-infected mice.

In an effort to determine the underlying mechanisms by which MTF alleviates hepatic pathology, the redox status, TGF-β, and FGF21 levels in the hepatic tissues were measured. In comparison to the positive control groups, the MTF, and combined groups exhibited a significant reduction in oxidative stress, as evidenced by lower levels of MDA and NO and higher levels of SOD and catalase (Hou et al. 2010; Esteghamati et al. 2013; Ashabi et al. 2015). Several previous studies, such as Marchetti et al. (2004) and Kelly et al. (2015), have reported that MTF lowered levels of MDA by inhibiting the mitochondrial complex-I of the respiratory chain and reducing the formation of reactive oxygen species. Additionally, it could halt the production of reactive nitrogen species like NO at the intracellular level (Esteghamati et al. 2013).

NO is a highly reactive chemical that plays a significant role in hepatic cell damage. Different cells, including hepatocytes, kupffer cells, macrophages, and endothelial cells, secrete it in the liver (Aksu et al. 2010). Fan et al. (2004) earlier established the role of NO in the pathophysiology of *T. canis*-induced hepatic disease. Therefore, inhibiting NO production could aid in mitigating the damage caused by *T. canis* infection. This finding was demonstrated by Espinoza et al. (2002), who reported that inhibition of NO production significantly reduced lung pathology in *T. canis*-infected rats.

Fibrosis is considered to be primarily regulated by TGF-β signaling. According to Zhang (2017) and Mi et al. (2019), it controls multiple signaling pathways contributing to liver fibrosis. Additionally, FoxO3a (Forkhead box O3) and the DNA demethylase TET3 (Tet methylcytosine dioxygenase 3) are upregulated by TGF-β in hepatic stellate cells (HSC), which promotes hepatic fibrogenesis (Xu et al. 2020; Kim et al. 2021). Therefore, the inactivation of TGF-β prevents HSC activation and liver fibrosis (Fan et al. 2019).

In contrast, FGF21 is predominantly expressed in hepatocytes, and its administration as a treatment has multiple direct and indirect effects on the liver (Coskun et al. 2008). It has anti-steatotic and anti-inflammatory properties (Keinicke et al. 2020; Seitz and Hellerbrand 2021). It also inhibits HSC activation via TGF-β and NF-κB pathways, and it can induce HSC apoptosis through caspase-3, thereby reducing hepatic fibrogenesis (Xu et al. 2016). FGF21 ameliorates hepatic fibrosis by multiple mechanisms, such as inhibition of TGF-β (Meng et al. 2021).

In the present study, we examined changes in the expression of TGF β-mRNA and FGF21 levels in the liver of *T. canis*-infected mice. TGF-β mRNA expression was enhanced in the infected untreated mice compared to the negative control. In acute and chronic phases of infection, Its expression was significantly decreased under the effect of combination therapy compared to the groups treated with either ABZ or MTF alone. These results are consistent with Li et al. (2015) and Lu et al. (2015), who illustrated that MTF had inhibitory effects on the TGF β signaling pathway.

Regarding levels of FGF21 in the current study, all treatment groups showed significant increases compared to the positive control groups. MTF and combined treated groups showed the highest levels. As previously reported by Nygaard et al. (2012), metformin is a potent inducer of FGF21 expression in primary rat and human hepatocytes through activating 5′ adenosine monophosphate-activated protein kinase (AMPK). Based on the previously reported results, we could attribute the significant improvement in hepatic pathology in the groups that received MTF or combined treatment in the present study to the potent anti-oxidant effects of MTF together with its effects in inhibition of TGF-β expression and enhancement of FGF21 expression.

During the course of *T. canis* infection, the larvae travel across all body organs. As the infection progresses, they tend to leave the different organs, such as the liver and lungs, and frequently build up in the muscles and the brain (Chou et al. 2017). According to Holland and Hamilton (2013), the brain is seen as a sanctuary for these larvae, protecting them from the host’s immunological response, which aligns with the findings of this work. As larval counts in the positive control group’s brain and muscles increased in the infection's chronic phase compared to the acute phase. However, their counts in the liver in the chronic phase were significantly lower than in the acute phase.

Previous research on *T. canis* infection has shown that the absence of inflammation around the larvae in the brain is a common observation in the mouse model (Liao et al. 2008; Eid et al. 2015; Chou et al. 2017). In the same context, Resende et al. (2015) reported the same observation, but they stated the presence of hemorrhagic areas as a sign of active larval movement in the brain. Furthermore, Othman et al. (2010) reported no discernible inflammatory response around the larvae. However, they identified the existence of vascular congestion close to some larvae. In the current study, we have noticed similar results indicating clogged blood arteries close to the larvae inside the positive control group.

The present investigation revealed that infection with *T. canis* resulted in a significant increase in the levels of TNF-α inside the brain tissues of the infected mic. Similarly, Othman et al. (2010) showed upregulation of the expression of TNF-α mRNA in the brain of *T. canis*-infected mice with the progression of the infection. TNF-α is a pro-inflammatory cytokine that is released by a wide variety of brain cells such as neurons, astrocytes, microglia, and oligodendrocytes. Its receptors are distributed throughout the CNS (Probert 2015). Its expression is typically increased in numerous CNS diseases. It has been proved that TNF-α has a significant role in the pathogenesis of parasitic infections affecting the brain such as African trypanosomiasis and cerebral malaria (Kinra and Dutta 2013; Kitwan et al. 2023).

Although increased production of pro-inflammatory cytokines, such as TNF-α, is helpful in boosting clearance of invasive infections and phagocytosis of cell debris, prolonged inflammatory processes can cause harmful brain damage and neurodegeneration (Lucas et al. 2006; Becher et al. 2017). Our results showed a significant reduction in TNF-α levels in the brains of mice that received MTF or combined treatment. MTF was previously reported to induce neuroprotective effects on traumatic brain injury in rats via inhibiting microglial activation and decreasing TNF-α production in the brain (Tao et al. 2018). The existing body of evidence indicates that MTF exhibits robust neuroprotective properties when used to treat various neurological disorders, such as brain ischemia (Zhang et al. 2022), traumatic brain injury (Tao et al. 2018), an Alzheimer’s disease model induced by streptozotocin (Saffari et al. 2020), and brain injury induced by sepsis (Tang et al. 2017).

A comprehensive investigation was conducted to examine the function of MMP-9 in the etiology of various neurological diseases. For instance, previous studies have shown its involvement in several types of brain traumas, including traumatic, hemorrhagic, and ischemic injuries (Chaturvedi and Kaczmarek 2014; Shao et al. 2014; Pijet et al. 2018). Moreover, it is well acknowledged that it serves as a reliable marker for neuroinflammation in different central nervous system disorders, including viral and bacterial meningitis as well as multiple sclerosis (Sulik and Chyczewski 2008; Aung et al. 2015).

In the present research, an investigation was conducted to compare the expression of MMP-9 in the brain of mice across various study groups. The results revealed a substantial increase in MMP-9 expression in the positive control group compared to the negative control group. A decrease in expression was seen in mice subjected to treatment with either MTF or a combination of treatments, regardless of whether it was during the acute or chronic stages of the infection. The findings of this study demonstrate the significant impact of MTF on the expression of MMP-9. Prior research conducted by Zhang et al. (2017) demonstrated a decrease in the expression of MMP-9 after the injection of MTF in the treatment of traumatic brain injury.

Migration of *Toxocara canis* larvae induces dynamic changes in the systemic immune response of the host. In the current study, *T. canis* infection induced a predominant decrease in IL-12 and IFN-γ levels and an increase in IL-4 along the course of the infection. Additionally, IL-10 levels increased significantly more during the chronic phase than during the acute phase. These findings are supported by prior research (Kuroda et al. 2001; Resende et al. 2015; Ruiz-Manzano et al. 2019). The findings of this research suggest that the immune mediators’ expression pattern was altered by the administration of MTF or combined treatment. There was an increase in levels of IL-12 and IFN-γ and a reduction in IL-4 and IL-10 levels. The results presented in this study align with the previous research conducted by Wang et al. (2020) and Nicolao et al. (2023), which also showed similar effects of MTF.

*T. canis* larvae use crucial immunological mechanisms, including the suppression of IL-12 and IFN-γ, as survival mechanisms. This suppression hinders the recruitment of macrophages and prolongs the lifespan of the parasite (Kuroda et al. 2001). Moreover, increased IL-10 expression is another contributor to the development and extension of toxocariasis pathology (Othman et al. 2011). Findings suggest that immunomodulation is involved in MTF’s protection against toxocariasis. The increase in IL-12 levels under the influence of MTF suggests that this drug prevents *T. canis* from inhibiting IL-12 and contributes to the body’s anti-infection response. This result is consistent with previous research indicating that an increase in IL-12 and IFN-γ may enhance the protective host response against *T. canis* (Pilarczyk et al. 2008; de Avila et al. 2016). The elevated IL-12 levels and decreased infection development in the MTF or combined treated groups also increased. This finding may have been due to the MTF-induced suppression of IL-10 expression, as both IL-10 and IL-12 have an antagonistic interaction (Torina et al. 2005; de Avila et al. 2016).

## Conclusion

Our findings indicate that MTF has a positive effect on reducing the pathological changes caused by *T. canis* infection in mice. It decreased recovery in the liver, brain, and muscles of developing larvae. Combined therapy was superior to ABZ alone in treating hepatic and cerebral pathologies. In addition, MTF was able to regulate the host’s immune response and oppose the immunomodulatory processes induced by *T. canis* infection. Identical effects were observed during both the acute and chronic phases of infection. We hypothesize that metformin exerts its anti-toxocara effects by modulating the immune response and ameliorating infection-related pathology. Therefore, MTF should be considered an adjuvant therapy for the treatment of experimental toxocariasis.

## Data Availability

Not applicable.

## Abbreviations

*T. canis*: *Toxocara canis*
ABZ: Albendazole
MTF: Metformin
PI: Post-infection
HCL: Hydrochloric acid
H&E: Hematoxylin and eosin
MMP-9: Matrix metalloproteinase
NO: Nitric oxide
MDA: Malondialdehyde
SOD: Superoxide dismutase
ELISA: Enzyme-linked immunosorbent assay
FGF21: Fibroblast growth factor 21
TNF-α: Tumor necrosis factor-alpha
IL-12: Interleukin-12
IFN-γ: Interferon-gamma
IL-4: Interleukin-4
IL-10: Interleukin-10
qRT-PCR: Quantitative real-time polymerase chain reaction
TGF-β: Transforming growth factor β
